# Adapting and Validating Tools to Assess the Usability and Acceptability of mHealth Tools Among Community Health Workers in Rural Settings: Development and Usability Study

**DOI:** 10.2196/64916

**Published:** 2026-02-20

**Authors:** Jonathan Nkurunziza, Sarah Nuss, Eve Hiyori Estrada, Marthe Kubwimana, Adeline Adwoa Boatin, Laban Bikorimana, Richard Ribon Fletcher, Nissi Byiringiro, Bethany Hedt-Gauthier, Vincent Kalumire Cubaka

**Affiliations:** 1 Partners In Health, Rwanda Kigali Rwanda; 2 Program in Global Surgery and Social Change Harvard Medical School Boston, MA United States; 3 Warren Alpert Medical School Brown University Providence, RI United States; 4 Department of Global Health and Social Medicine Harvard Medical School Boston, MA United States; 5 Department of Obstetrics and Gynecology Massachusetts General Hospital Boston, MA United States; 6 Department of Mechanical Engineering Massachusetts Institute of Technology Cambridge, MA United States; 7 Insightiv LTD Kigali Rwanda; 8 University of Global Health Equity, Burera, Rwanda, Department of Community Health and Social Medicine Kigali Rwanda

**Keywords:** mobile health, digital health, Africa, global surgery, community health workers, adaptation and validation, usability, acceptability

## Abstract

**Background:**

Mobile health (mHealth) apps are increasingly leveraged to support community health workers (CHWs) in delivering high-quality care, particularly in low- and middle-income countries. However, despite the proliferation of mHealth tools, few have been implemented at scale, partly due to limited attention to usability and acceptability among end users. In sub-Saharan Africa, mHealth tools designed for CHWs often lack systematic evaluation using validated instruments tailored to local contexts. Without such assessments, it is difficult to ensure that these tools can be integrated effectively into CHW workflows and scaled sustainably.

**Objective:**

This study aimed to adapt and validate existing mHealth usability and acceptability assessment tools to be contextually appropriate for CHWs in Rwanda. Specifically, we sought to ensure contextual appropriateness for CHWs supporting postoperative home follow-up for women after cesarean delivery. The resulting tool was designed for use in an implementation study of a novel CHW-led mHealth app.

**Methods:**

This study was conducted in the Kirehe district, Rwanda, from October 2022 to March 2023. We adapted 2 established tools—the mHealth App Usability Questionnaire and selected items from the Practitioner Opinion (Acceptability) Scale—and added new items that reflect core functions of the CHW-focused mHealth app. All items were translated into Kinyarwanda and simplified to align with CHWs’ educational levels. We conducted a three-stage validation that consisted of (1) content validity testing with 8 local and international experts using a recommended content validity index threshold of >0.78; (2) face validity testing with 10 CHWs using a recommended face validity index threshold of ≥0.60; and (3) reliability testing using responses from 30 CHWs, with a Cronbach α coefficient of ≥0.70 indicating acceptable internal consistency.

**Results:**

Of the 25 items assessed, 22 (88%) achieved a content validity index score of >0.78 for both clarity and relevance. The face validity index across all 22 items was 0.991, indicating strong comprehensibility and relevance to CHWs. Internal consistency was high: the Cronbach α was 0.86 for the mHealth App Usability Questionnaire items, 0.73 for the Practitioner Opinion (Acceptability) Scale items, and 0.87 for the newly developed questions. The final tool—named the Community Health Worker mHealth Usability and Acceptability Assessment Tool—included 22 items with strong content validity, face validity, and internal reliability.

**Conclusions:**

This study presents a rigorously adapted and validated tool for assessing mHealth usability and acceptability among CHWs in Rwanda. The Community Health Worker mHealth Usability and Acceptability Assessment Tool can guide future evaluations of mHealth interventions in similar contexts and serve as a model for localizing mHealth assessment tools in low- and middle-income country settings to ensure fit-for-purpose implementation.

## Introduction

Mobile health (mHealth) apps have emerged as important tools to support health care workers in delivering high-quality care, particularly in low- and middle-income countries. Over the past 2 decades, an increasing number of mHealth solutions have been developed to assist community health workers (CHWs) in performing critical tasks across a range of health interventions [1,2]. CHWs, who often have limited formal training, nonetheless play a vital role in screening, diagnosing, and managing health conditions at the community level [3]. mHealth apps targeting CHWs are typically designed to support specific workflows or interventions. However, to ensure that these tools are adopted and used effectively, it is essential to evaluate their usability and acceptability among intended users. Robust assessment can guide the refinement of interface design and core functionalities, ultimately improving user experience and the quality of care delivered. Despite the increasing deployment of CHW-focused mHealth tools, few published studies have quantitatively assessed their usability or acceptability in sub-Saharan Africa (SSA) [4-6], and to our knowledge, there are no validated quantitative tools that have been developed for this purpose.

There are several tools that assess the usability of digital interventions or mHealth apps among target users outside of CHWs. Examples of usability assessment questionnaires geared toward patients include the Mobile App Rating Scale, a tool to measure the quality of apps commonly used in the health domain [7]; the System Usability Scale, developed to assess the usability of health care innovations [8]; and the Health Information Technology Usability Evaluation Scale, a tool used to assess the usability of mHealth technologies in groups of adults living with chronic diseases [9]. Tools that assess usability among clinical providers are the Telehealth Usability Tool [10] and the mHealth App Usability Questionnaire (MAUQ) [11]. The Telehealth Usability Tool was designed to evaluate the interface quality, reliability, and satisfaction of computer-based telehealth videoconferencing software, whereas the MAUQ was developed to evaluate mHealth apps.

Evaluation of user acceptability is also a critical aspect in the development of mHealth apps. The Practitioner Opinion (Acceptability) Scale (POAS) is an example of a tool designed to evaluate the acceptability of decision aids during the development process and early evaluation stages [12]. Other examples of acceptability tools to evaluate mHealth technology include the Acceptability E-scale, [13], the technology acceptance model [14], and the User Engagement Scale [15]. However, none of these were developed to assess acceptability among CHWs.

In this paper, we describe our process of adapting existing tools to develop and validate a quantitative tool to assess CHWs’ acceptability of an mHealth app and its usability. This validated tool was specifically developed to assess the usability and acceptability of a new mHealth app to support CHW-led comprehensive home-based follow-up care among women who had delivered via cesarean section in rural Rwanda.

## Methods

### Study Design

Between October 2022 and March 2023, we adapted and validated a tool to assess the usability and acceptability of a novel mHealth screening app in a sample of target end users (CHWs). The mHealth app supports comprehensive home-based follow-up care among women who have delivered via cesarean section. The usability and acceptability assessment tool we developed is referred to as the Community Health Worker mHealth Usability and Acceptability Assessment Tool (CHW-MUAAT).

### Study Setting

This study was implemented in the Kirehe district located in Eastern Province, rural Rwanda. The Kirehe district is represented by Kirehe District Hospital, with 19 health centers and 2448 CHWs. This hospital operates under the Rwanda Ministry of Health and is supported by Inshuti Mu Buzima, a Rwandan sister organization of the US-based nongovernmental organization Partners In Health since 2005. This study was conducted as part of the study titled “mHealth - community health worker tool for comprehensive postcesarean follow-up in rural Rwanda,” which was being conducted in the same area.

### Selection of the Existing Tools and Initial Questions

After reviewing multiple existing tools to assess usability and acceptability of mHealth or digital health interventions, our team decided to move forward with 2 existing tools due to the relevant information they provided to our study. To assess usability among CHWs, we adapted the MAUQ for stand-alone mHealth apps, which was developed and validated in English by Zhou et al [11]. The original MAUQ comes in 4 versions depending on the type of app (interactive or stand-alone) and the target users (patients or health care providers). Given that our mHealth app is stand-alone and geared toward CHWs, we adapted the MAUQ for stand-alone mHealth apps used by health care providers. This version of the MAUQ includes 14 questions. For acceptability, we selected the questions most relevant to our study from the POAS, validated by O’Connor and Cranney [12]; we started with 4 questions that were deemed relevant out of the 15 from this tool. In addition, we developed and added 7 usability questions; these questions were drafted by the core study team members to reflect core functions of the mHealth screening app under development. These functions were not captured in the MAUQ or POAS questions and underwent the validation steps described in subsequent sections. To assess the reading level of our assessment tool, we intentionally designed all items to meet a below 8th‑grade reading level, following widely accepted best practices in health communication. This decision was guided by recommendations from the National Institutes of Health (NIH), which emphasize using plain language, short and clear sentences, and familiar vocabulary in survey instruments to promote comprehension and reduce respondent burden. These NIH guidelines support creating materials that are accessible across diverse literacy levels and help minimize misunderstanding or response error (NIH Plain Language Guidance [16]).

### Translation and Adaptation

After ensuring its comprehensibility with a reading level equivalent to sixth grade, the CHW mHealth usability and acceptability assessment tool underwent a cross-cultural adaptation process, which consisted of translation into Kinyarwanda, back translation, and harmonization, as described by Beaton et al [17]. A study team member fluent in Kinyarwanda and English translated the adapted questionnaire from English into Kinyarwanda. After translation, an independent translator with skills in the health domain and who did not have access to the original version of the assessment questions translated the questions back into English. The study team and translator met to review and complete a harmonization process to help identify any ambiguity, errors, or confusion in the translated version and ensure that the tool was appropriate for the cultural context.

### Validity and Reliability Testing

Validity testing comprised 2 assessments: content validity testing and face validity testing, whereas reliability was evaluated through internal consistency analysis.

#### Content Validity

Content validity assessed the relevancy and clarity of the tool among content experts. Eight content experts, including app developers, study team members, and clinicians, were conveniently asked to rate the relevancy and clarity of items from the CHW-MUAAT on a scale from 1 to 4 (1=“not relevant” or “not clear”; 4=“very relevant” or “very clear”). Content validity was established using the content validity index (CVI). The CVI was calculated using the number of experts who agreed that the item was relevant or clear (scores of 3 or 4) divided by the total number of experts. The scores for each individual item are referred to as the “item CVI.” On the basis of recommended CVI thresholds from the literature [18], items with an item CVI of >0.78 were deemed content valid and remained on the survey. Those with an item CVI score of ≤0.78 were removed.

#### Face Validity

Face validity was established by assessing the clarity and comprehensibility of the translated tool among target end users. In total, 10 target users (CHWs) from the Kirehe district were selected at random and asked to rate the clarity and comprehensibility of the translated items on the assessment tool on a scale from 1 to 4 (1=“not at all clear” or “not at all understandable”; 4=“very clear” or “very understandable”). The face validity index (FVI) was calculated by dividing the number of CHWs who agreed that the item was understandable (score of 3 or 4) by the total number of CHWs (n=10). On the basis of recommendations from the literature [19], an FVI above 0.74 was considered excellent, an FVI of 0.60 to 0.74 was considered good, and an FVI of 0.54 to 0.59 was considered fair. Items with an FVI of ≥0.60 were considered acceptable and kept.

#### Reliability Testing

After the content and face validity testing, the adapted tool was used to assess the usability and acceptability of the mHealth app among 30 CHWs. The 30 CHWs received explanations on the use of the mHealth CHW app and were asked to respond to all questions on the app during 3 sample patient vignette role-plays led by a study team facilitator. After the sample role-play, the CHWs were asked to answer questions from the CHW-MUAAT. The full results of this assessment are presented elsewhere. For this study, we used the data from reliability testing to assess the internal consistency of the tool. The internal consistency of the tool was evaluated by calculating the Cronbach α coefficient [20]. A higher α value suggests greater internal consistency reliability, with an α value greater than 0.70 deemed to indicate good reliability [21,22].

### Ethical Considerations

This tool adaptation and validation study falls under a larger study titled “mHealth - community health worker tool for comprehensive postcesarean follow-up in rural Rwanda,” which received human participant ethical review approval from the Rwanda National Ethics Committee (109/RNEC/2022) and the Institutional Review Board of the Harvard Faculty of Medicine (22-1025). For the reliability testing, all CHWs provided informed consent before participating in the mHealth usability and acceptability assessments and were able to opt out of participation in the study activities. Data used in measuring validity and reliability were deidentified. Participants were compensated with 6000 Rwandan francs (approximately US $4.15) for their time and travel related to the study.

## Results

The first adapted tool included 14 questions from the MAUQ, 4 POAS questions, and 7 questions added by the study team, yielding a total of 25 questions. All questions were modified for a sixth-grade reading level and underwent cross-cultural translation. The final questions for this first adaptation are presented in Table 1.

**Table 1:** Adaptation of original questions to sixth-grade reading level and cross-language translation.

Source and original question		Adapted question to sixth-grade English reading level (back translation)	Adapted question to Kinyarwanda
**MAUQ^a^**			
	“The app was easy to use.”	“The app is easy to use.”	“Iyi apurikasiyo iroroshye kuyikoresha.”
	“It was easy for me to learn to use the app.”	“It is easy for me to learn to use the app.”	“Biranyorohera kwiga gukoresha iyi apurikasiyo y’ikoranabuhanga.”
	“The navigation was consistent when moving between screens.”	“The navigation is consistent when moving between screens.”	“Kugenda mpinduranya aho nkorera ni ibintu bihamye cyangwa bikorwa mu byryo bumwe mu gukoresha iyi apurikasiyo.”
	“The interface of the app allowed me to use all the functions (such as entering information, responding to reminders, viewing information) offered by the app.”	“I can use all the functions (such as entering information, responding to reminders, viewing information) in the app.”	“Nshobora gukoresha fongisiyo zose / ibice byose (nko kwinjiza amakuru, gusubiza ibijyanye no kwibutsa, kureba amakuru).”
	“Whenever I made a mistake using the app, I could recover easily and quickly.”	“Whenever I make a mistake on the app, it is easy and quick to correct it.”	“Igihe cyose nkoze ikosa cyangwa nibeshye mu gukoresha apurikasiyo, biroroshye kandi biruhuta kurikosora.”
	“I like the interface of the app.”	“I like the interface of the app.”	“Nakunze uburyo apurikasiyo igaragara mu kuyikoresha.”
	“The information in the app was well organized, so I could easily find the information I needed.”	“The information in the app is well organized, so I can easily find the information I need.”	“Amakuru yashyizwe neza muri apurikasiyo ku buryo nshobora kubonamo ayo nkeneye ku buryo bworoshye.”
	“The app adequately acknowledged and provided information to let me know the progress of my action.”	“The app does a good job of letting me know my progress.”	“Apurikasiyo imfasha kumenya aho ngeze nkora.”
	“The amount of time involved in using this app has been fitting for me.”	“The time it takes to use this app works well for me.”	“Umwanya bimfata mu gukoresha iyi apurikasiyo mu kazi urakwiye kuri njye.”
	“Overall, I am satisfied with this app.”	“Overall, I am satisfied with this app.”	“Muri rusange nyuzwe niyi apurikasiyo.”
	“The app improved my access to delivering healthcare services.”	“The app improves my ability to deliver healthcare services.”	“Apurikasiyo itezimbere ubushobozi bwanjye bwo gutanga serivisi z’ubuzima.”
	“The app helped me manage my patients’ health effectively.”	“The app helps me manage my patients’ health effectively.”	“Apurikasiyo imfasha mu kwita neza ku buzima bw’abarwayi banjye mu buryo buboneye.”
	“This app has all the functions and capabilities I expected it to have.”	“This app has all the functions and capabilities I expected it to have.”	“Iyi apurikasiyo ifite imikorere n’ubushobozi nateganyaga ko ifite.”
	“I could use the app even when the Internet connection was poor or not available.”	“I can use the app even when the Internet connection was poor or not available.”	“Nshobora gukoresha apurikasiyo nubwo umurongo wa interineti waba ari muke cyangwa ari ntawo.”
**POAS^b^**			
	“It will be easy for me to understand how to use the app.”	“It is easy for me to understand how to use the app.”	“Biranyoroheye kumva uburyo bwo gukoresha apurikasiyo.”
	“This strategy is compatible with the way I think things should be done.”	“The app is a good fit for the way I conduct visits to women after cesarean section.”	“Apurikasiyo ikwiranye neza n’uburyo bwo gusura abagore nyuma yo kubyara babazwe.”
	“Using this strategy will save me time.”	“The app will save me time during home visits to women who delivered by cesarean section.”	“Apurikasiyo izamfasha kuzigama igihe nakoreshaga mugihe cyo gusura mu rugo ababyeyi babyaye babazwe.”
	“There is a high probability that using this strategy may cause or result in more benefit than harm.”	“I think using this mHealth app will be more helpful than hurtful.”	“Ndatekereza ko gukoresha iyi apurikasiyo ya mHealth bizafasha cyane kuruta guteza ibyago.”
**Questions added by the study team members**			
	—^c^	“This app would be useful for my home visits to women after cesarean section.”	“Iyi apurikasiyo yaba ingirakamaro mu gusura mu rugo ababyeyi nyuma yo kubyara babazwe.”
	—	“It is easy for me to log into the app with my username and password.”	“Biranyorohera kwinjira muri apurikasiyo ukoresheje izina ryanjye n’ijambo ryibanga.”
	—	“It is easy for me to find the correct patient using the patient name ID.”	“Biranyorohera kubona umurwayi nyawe nkoresheje ijambo riranga umurwayi.”
	—	“It is easy for me to take or retake a picture of the wound using the app.”	“Biranyorohera gufata ndetse no kongera gufata ifoto y'igisebe nkoresheje apurikasiyo.”
	—	“It is easy for me to read the recommendations given by the app.”	“Biranyorohera gusoma amabwiriza yatanzwe na apurikasiyo.”
	—	“It is easy for me to communicate with women when using the app.”	“Biranyorohera kuvugana n'mubyeyi mugihe nkoresheje apurikasiyo.”
	—	“I feel comfortable using the app during visit for follow-up of women who delivered by C-section.”	“Ndumva mbohokewe cyangwa nifitiye ikizere mu gukoresha iyi apurikasiyo mugihe cyo gusura mu rugo kugirango nkurikirane abagore babyaye babazwe.”

^a^MAUQ: mHealth App Usability Questionnaire.

^b^POAS: Practitioner Opinion (Acceptability) Scale.

^c^Not applicable.

A total of 10 content experts participated in the content validity survey. In total, 88% (22/25) of the items received an item CVI score of >0.78 on both the clarity and relevance scales (Table 2). Three items had an item CVI lower than this threshold and were removed. The 3 items that were removed from the first adapted set of questions were “The app does a good job of letting me know my progress,” “The navigation is consistent between screens,” and “I can use all the functions (such as entering information, responding to reminders, and viewing information) in the app.”

All the remaining 22 items received individual FVI scores of >0.60 for clarity and relevance and, thus, were retained in the full final tool (Table 2). The FVI score for clarity across all questions was 0.991 and was the same for relevance. The full final tool is available in Table 3. The Cronbach α value was 0.86 for the 12 MAUQ usability questions, 0.73 for the 4 POAS acceptability questions, and 0.87 for the questions added by the study team, indicating the high internal consistency reliability of these questions.

**Table 2:** Content and face validity testing of items considered for the Community Health Worker mHealth Usability and Acceptability Assessment Tool.

Item	Content validity testing with content expert panel^a^			Face validity testing with CHWs^b,c^	
	Clarity I-CVI^d^	Relevance I-CVI	Clarity FVI^e^		Comprehension FVI
“The app is easy to use.”	1	1	1		1
“It is easy for me to learn to use the app.”	0.9	1	1		1
“The navigation is consistent when moving between screens.”	0.6	0.8	—^f^		—
“I can use all the functions (such as entering information, responding to reminders, viewing information) in the app.”	0.6	0.9	—		—
“Whenever I make a mistake on the app, it is easy and quick to correct it.”	0.9	1	1		1
“I like the interface of the app.”	0.9	0.9	1		1
“The information in the app is well organized, so I can easily find the information I need.”	0.9	1	1		1
“The app does a good job of letting me know my progress.”	0.9	0.7	—		—
“The time it takes to use this app works well for me.”	0.9	1	1		1
“Overall, I am satisfied with this app.”	1	0.9	1		1
“The app improves my ability to deliver healthcare services.”	1	1	1		1
“The app helps me manage my patients’ health effectively.”	0.9	0.9	1		1
“This app has all the functions and capabilities I expected it to have.”	1	1	1		1
“I can use the app even when the Internet connection was poor or not available.”	0.8	1	0.8		0.8
“This app would be useful for my home visits to women after cesarean section.”	1	0.9	1		1
“It is easy for me to log into the app with my username and password.”	1	1	1		1
“It is easy for me to find the correct patient using the patient name ID.”	1	1	1		1
“It is easy for me to take or retake a picture of the wound using the app.”	1	1	1		1
“It is easy for me to read the recommendations given by the app.”	1	1	1		1
“It is easy for me to communicate with women when using the app.”	0.9	1	1		1
“I feel comfortable using the app during home visit for follow up of women who delivered by caesarean section.”	1	1	1		1
“It is easy for me to understand how to use the app.”	1	0.8	1		1
“The app is a good fit for the way I conduct visits to women after cesarean section.”	0.8	0.9	1		1
“The app will save me time during home visits to women who delivered by cesarean section.”	1	1	1		1
“I think using this mHealth app will be more helpful than hurtful.”	0.9	0.9	1		1

^a^In total, 10 content experts from the study team participated in content validity testing. Items with a clarity item content validity index or relevance item content validity index of <0.78 were removed from the final tool.

^b^In total, 10 community health workers participated in face validity testing. Items with a face validity index of <0.60 were removed from the final tool.

^c^CHW: community health worker.

^d^I-CVI: item content validity index.

^e^FVI: face validity index.

^f^Not applicable.

**Table 3:** Contents of the Community Health Worker mHealth Usability and Acceptability Assessment Tool^a^.

Item number	Statement	“Strongly disagree” score	“Disagree” score	“Neutral” score	“Agree” score	“Strongly agree” score
1	“The app is easy to use.”	1	2	3	4	5
2	“It is easy for me to learn to use the app.”	1	2	3	4	5
3	“Whenever I make a mistake on the app, it is easy and quick to correct it.”	1	2	3	4	5
4	“I like the interface of the app.”	1	2	3	4	5
5	“The information in the app is well organized, so I can easily find the information I need.”	1	2	3	4	5
6	“The time it takes to use this app works well for me.”	1	2	3	4	5
7	“Overall, I am satisfied with this app.”	1	2	3	4	5
8	“The app improves my ability to deliver healthcare services.”	1	2	3	4	5
9	“The app helps me manage my patients’ health effectively.”	1	2	3	4	5
10	“This app has all the functions and capabilities I expected it to have.”	1	2	3	4	5
11	“Once I have logged in, I can use the app even when the Internet connection was poor or not available.”	1	2	3	4	5
12	“This app would be useful for my home visits to women after cesarean section.”	1	2	3	4	5
13	“It is easy for me to log into the app with my username and password.”	1	2	3	4	5
14	“It is easy for me to find the correct patient using the Patient name ID.”	1	2	3	4	5
15	“It is easy for me to take or retake a picture of the wound using the app.”	1	2	3	4	5
16	“It is easy for me to read the recommendations given by the app.”	1	2	3	4	5
17	“It is easy for me to communicate with women when using the app.”	1	2	3	4	5
18	“I feel comfortable using the app during home visit for follow up of women who delivered by caesarean section.”	1	2	3	4	5
19	“It is easy for me to understand how to use the app.”	1	2	3	4	5
20	“The app is a good fit for the way I conduct visits to women after cesarean section.”	1	2	3	4	5
21	“The app will save me time during home visits to women who delivered by cesarean section.”	1	2	3	4	5
22	“I think using this mHealth app will be more helpful than hurtful.”	1	2	3	4	5

^a^Questions 1 to 12 are from the mHealth App Usability Questionnaire (MAUQ). Questions 13 to 18 were developed to assess the usability of core functions of the mobile health community health worker app not captured by the MAUQ or Practitioner Opinion (Acceptability) Scale (POAS). Questions 19 to 22 were adapted from the POAS.

## Discussion

### Principal Results

In this study, we adapted 2 existing tools, the MAUQ and POAS, to evaluate CHWs’ perceptions of the usability and acceptability of a novel mHealth app for comprehensive home-based follow-up care among women who have delivered via cesarean section in Rwanda. Our final tool included 22 items in total: 12 (55%) out of the 15 original items from the MAUQ tool, all 4 (18%) of the items that we pulled from the POAS, and 6 (27%) new items added by our study team. The final items had high content validity among content experts and high face validity and internal reliability among CHWs.

While there is an increasing number of mHealth apps designed to support CHWs across SSA, very few are operating at a large scale and beyond isolated research settings [2,23,24]. One of the key barriers to scale-up is the limited understanding of whether these tools are perceived as usable and acceptable by the CHWs expected to adopt them. Without sufficient usability and acceptability, even well-designed tools are unlikely to integrate effectively into CHW workflows. Although a few studies in SSA have examined these dimensions among CHWs [2,4-6], most have not used adapted or validated tools, limiting the reliability and comparability of their findings. A recent review found that 70% of researcher-developed usability questionnaires for mHealth apps were not validated—and those validated were not tailored to the community health context [25]. Validated assessment frameworks are critical to draw robust conclusions about user experience and determining the potential for mHealth tools to be well integrated into routine practice.

This quantitative CHW-MUAAT scale provides a standardized, scalable way to summarize usability and acceptability constructs, which facilitates comparison of potential digital tools in one environment or of the functionality of a single tool in different contexts [26]. However, complementary qualitative data can offer deeper insights into the contextual factors that shape CHW perceptions of usability or acceptability. For example, a mixed methods study identified that CHWs in Uganda had high rates of digital health acceptance but low digital tool use due to low smartphone ownership [27]; similarly, a study of *YendaNafe*, a mobile app to support CHW activities in Malawi, found an overall positive acceptance of the app but that there were several facilitators of and barriers to actual app use [28]. For this study, our qualitative results indicated general acceptability and emphasized that strong CHW-patient relationships and trust were needed for successful implementation (full results available elsewhere [29]).

To meaningfully inform the integration of an mHealth app into CHWs’ workflows, usability and acceptability assessments must be tailored to the local context and to the needs and capacities of end users. In our study, this required careful linguistic and cognitive adaptation of existing measurement tools. Given that most CHWs in Rwanda have completed only primary school education, our team revised the wording of several items to ensure that they were easily understandable. For example, the original item “There is a high probability that using this strategy may cause or result in more benefit than harm” was simplified to “I think using this mHealth app will be more helpful than hurtful” [30]. Additionally, all items were translated into Kinyarwanda, the primary language spoken by CHWs, with attention to clarity and cultural appropriateness.

Beyond linguistic adaptation, we also modified the content of existing tools to better capture usability and acceptability as related to the specific task—home-based follow-up care after cesarean delivery. This included rewording items from the MAUQ and POAS tools and adding step-specific questions relevant to CHWs’ real-world experience. For example, questions about ease of logging into the app were added given that many CHWs have limited experience with smartphones. This consideration is important in Rwanda, where, as of 2024, although 80% of households own a mobile phone, only 34% own a smartphone [31]. By demonstrating a structured approach to adapting and contextualizing usability and acceptability assessment tools, this study offers both a practical instrument and a replicable methodology for future CHW-focused mHealth evaluations in similar settings across the region.

This instrument can be adapted for other teams interested in assessing the usability and acceptability of a particular CHW mHealth tool with appropriate adaptation and validation. First, they may need to identify usability questions tailored to community health interventions, similarly to how our team added 7 usability questions specific to our postcesarean monitoring app. These questions will need to undergo reliability and validity assessments. The full tool should then be piloted in their settings, whereas smaller sample sizes may be warranted given the baseline work presented in this paper.

### Limitations

We acknowledge that the adaptation and validation of the tool has some limitations that should be considered. First, this study had a small sample size of content experts and CHWs. Because of the overall high scores and consistent conclusions, we are confident in the validity of the final tool developed, but future validation studies will likely need larger sample sizes if there is more variability in participant responses. Second, this tool focuses on a quantitative assessment that cannot pick up nuanced responses. Our final evaluation includes the quantitative responses collected using this tool during the usability and acceptability testing of the app. However, we do believe that these standardized assessments can help those evaluating mHealth apps identify areas for improvement and test against prespecified targets to determine whether the mHealth app is ready for broader use. Finally, this tool was designed for a specific intervention—a mobile phone app developed for CHWs to use during the home visit follow-up of women who delivered via cesarean section—and was only validated in the Kinyarwanda language. Other research groups should proceed cautiously and likely conduct their own validation studies when applying this tool to a different intervention or in a different language.

### Conclusions

Our team is using the final tool as a core component of evaluating an mHealth app used by CHWs to support women after cesarean delivery. The processes and tool described in this paper serve as a model for others conducting CHW usability and acceptability assessments of mHealth apps in SSA. Such assessments will be critical for moving toward better integration of innovative solutions into CHW activities.

## Data Availability

The datasets generated or analyzed during this study are not publicly available due to restrictions on data sharing in accordance with the Rwanda Data Protection and Privacy Law but are available from the corresponding author on reasonable request.

## Abbreviations

CHW: community health worker
CHW-MUAAT: Community Health Worker mHealth Usability and Acceptability Assessment Tool
CVI: content validity index
FVI: face validity index
MAUQ: mHealth App Usability Questionnaire
mHealth: mobile health
NIH: National Institutes of Health
POAS: Practitioner Opinion (Acceptability) Scale
SSA: sub-Saharan Africa
